# Death of Dr. Wainwright from the Bite of a Rattlesnake

**Published:** 1848-01

**Authors:** 


*Death of Dr. Wainwright from the Bite of a Rattlesnake.*—The melancholy death of Dr. Wainwright, from the bite of a snake, we have already recorded, but the circumstances attending the case are so fully set forth in the following, from the "Globe," that we lay it before our readers.—*Penn. Inq.*

On Thursday afternoon, Dr. W. received from a brother-in-law in Alabama, through one of our packets, a number of rare plants, &c. —the productions of that section of the Union—and, probably, for the purpose of furnishing a subject for more scientific experiments, a rattlesnake, six feet long, was contained in the invoice.

The reptile was securely boxed; but it seems that Dr. W., for the purpose of exhibiting it to some friends in the evening, took the box to the Broadway House, corner of Grand and Broadway, where, knocking off the top, the snake was let loose upon the bar-room floor. Throwing itself into a coil, the dangerous creature commenced that low hum, or species of ringing, (not the rattle,) which is peculiar to the species, and seemed inclined to remain quiet; probably the change of climate produced a sort of torpor, and it was repeatedly teased with a stick without betraying much viciousness. Indeed, one gentleman ventured so far as to raise it with the toe of his boot! escaping unscathed.

After being exposed some twenty minutes to the gaze of those present, Dr. Wainwright attempted to return the snake to the box, and for that purpose inconsiderately seized the venomous thing with his NAKED HAND! when, in an instant, with only the slightest premonitory rattle, the reptile raised his head, threw back his jaw, and STRUCK—the fangs entering between the fingers, and fastening on the inside of the ring-finger on the right hand!

Immediate measures were taken to prevent the spread of the poison through the system. The flesh in the neighbourhood of the part was cut out, and Dr. Wainwright removed to his house in Crosby street, where other medical and surgical aid was called, without delay, and in a few minutes the room was filled with his professional friends, among whom were Drs. Whittaker, Parker and Caldwell, of the Institute.

Energetic means were made use of to counteract the effect of the venom, but unaccountably, all known remedies seemed to be of no avail, and the entire arm commenced swelling most fearfully. At this juncture we are informed, that Dr. Wainwright, with much presence of mind, begged to have an amputation of the whole arm performed, but, after consultation, this course was deemed inadvisable, and the victim, enduring the most excruciationg agony, continued to sink, and finally expired at half an hour after midnight—the lamp of life going out at last quietly, and with apparently no struggle.

The unhappy man seemed to possess his full faculties almost to the last moment, and was perfectly aware of the fate to which he was inevitably hastening. Some fifteen minutes before his decease, turning to a friend who was supporting him, "This is horrible!" said he, as he felt the extreme pain leaving his hand, and the sensation of ease slowly creeping up the arm from the seat of the wound —"This is horrible!—to know that death is gradually feeling his way to my vitals!—That arm is dead already! and"—placing the uninjured hand over his heart—"the destroyer will soon be HERE !!" This acute knowledge of his sure dissolution, which, as a medical man, he must have possessed, could have been nought else than truly fearful.

The body after death, presented the usual appearance of disease from the bite of these hideous reptiles, it being frightfully swollen and mottled.

The snake, we believe, was secured by Mr. Martin, the proprietor of the Broadway House, by throwing a net over it, and has been killed.

Dr. Wainwright, we learn, was a native of England, and the son of one of the principal bankers in the British metropolis. He had been a resident of this city for some years, and had an extensive practice in addition to the position he occupied at the Crosby street Institute. He was 36 years of age, and has left a wife and two children, with a large circle of friends, to mourn his early and most agonizing death.

We learn that the body of the unfortunate man was embalmed, and will be buried in the Greenwood Cemetry on Sunday next.

